# Differences in Hematological Traits between High- and Low-Altitude Lizards (Genus *Phrynocephalus*)

**DOI:** 10.1371/journal.pone.0125751

**Published:** 2015-05-08

**Authors:** Songsong Lu, Ying Xin, Xiaolong Tang, Feng Yue, Huihui Wang, Yucheng Bai, Yonggang Niu, Qiang Chen

**Affiliations:** Institute of Biochemistry and Molecular Biology, School of Life Science, Lanzhou University, Lanzhou, China; University of Michigan, UNITED STATES

## Abstract

*Phrynocephalus erythrurus* (Lacertilia: Agamidae) is considered to be the highest living reptile in the world (about 4500-5000 m above sea level), whereas *Phrynocephalus przewalskii* inhabits low altitudes (about 1000-1500 m above sea level). Here, we report the differences in hematological traits between these two different *Phrynocephalus* species. Compared with *P*. *przewalskii*, the results indicated that *P*. *erythrurus* own higher oxygen carrying capacity by increasing red blood cell count (RBC), hemoglobin concentration ([Hb]) and hematocrit (Hct) and these elevations could promote oxygen carrying capacity without disadvantage of high viscosity. The lower partial pressure of oxygen in arterial blood (PaO_2_) of *P*. *erythrurus* did not cause the secondary alkalosis, which may be attributed to an efficient pulmonary system for oxygen (O_2_) loading. The elevated blood-O_2_ affinity in *P*. *erythrurus* may be achieved by increasing intrinsic O_2_ affinity of isoHbs and balancing the independent effects of potential heterotropic ligands. We detected one α-globin gene and three β-globin genes with 1 and 33 amino acid substitutions between these two species, respectively. Molecular dynamics simulation results showed that amino acids substitutions in β-globin chains could lead to the elimination of hydrogen bonds in T-state Hb models of *P*. *erythrurus*. Based on the present data, we suggest that *P*. *erythrurus* have evolved an efficient oxygen transport system under the unremitting hypobaric hypoxia.

## Introduction

Animals living in high altitude habitats have to manage certain additional physiological challenges in conditions of reduced oxygen availability and low ambient temperature. Matching O_2_ supply (inspired air) with O_2_ demand (tissue mitochondria) is necessary and important for both high-altitude natives and animals to acclimate to high altitude [1–3].

To live under high-altitude hypoxia, animals usually adopt some strategies or adjustments in the oxygen transport system. These adjustments should include at least three aspects [1]. Firstly, the highly efficient pulmonary ventilation and pulmonary O_2_ diffusion can help maintain high O_2_ partial pressures of arterial blood (PaO_2_). Pulmonary ventilation is mainly affected by the partial pressures of O_2_ and CO_2_ and the pH of arterial blood. These factors normally stimulate breathing via central and peripheral chemoreceptor [4–6]. Long time acclimatization to high altitude can relieve this hypoxic ventilatory response [5,7]. In addition, pulmonary O_2_ diffusion is mainly affected by the thickness and surface area of the pulmonary blood-gas interface [8–10]. Secondly, in order to ensure an adequate supply of O_2_ to the cells of aerobically metabolizing tissues, circulatory O_2_ delivery and tissue O_2_ diffusion can be enhanced by increasing the total cardiac output and the blood-O_2_ capacitance coefficient, such as by elevating hemoglobin concentration ([Hb]) and hematocrit (Hct) [1,11]. A moderately increased Hct is conducive to enhancing O_2_ carrying capacity of arterial blood, while an excessively increased Hct will result in the increase of blood viscosity to reduce the O_2_ carrying capacity by a higher peripheral vascular resistance and hence add more budgets for heart and blood circulation system [12–15]. Finally, fine-tuned adjustments in blood-O_2_ affinity are very important for O_2_ transport system during high altitude hypoxia. The regulation process may involve changes in intrinsic Hb-O_2_ affinity, the sensitivity of Hb to allosteric effectors and compensatory changes in concentration of allosteric effectors (particularly organic and inorganic anions) within the erythrocyte [1,16–19]. In addition, adaptive genetic variations of α- and β-like globin genes have also identified in many studies [20–24].

The Hb of jawed vertebrates is a heterotetramer which contains two α-globin chains and two β-globin chains with a heme group in each chain. During the process of vertebrate evolution, the α- and β-globin gene families have been subjected to repeated rounds of gene duplication and divergence [25,26]. Furthermore, studies have shown that the developmental regulation of Hb synthesis in some reptiles differ from other tetrapod vertebrates [27–29].

The mechanisms underlying the physiological acclimatization and genetic adaptation to high-altitude hypoxia have been studied extensively in birds and mammals. Although these mechanisms have also been found in some reptiles, how reptiles adapt to high-altitude hypoxia still remains largely unknown. Among over 40 species of Asian lizard genus *Phrynocephalus*, several phylogenetic independent lineages (*P*. *putjatia*, *P*. *vlangalii vlangalii*, *P*. *vlangalii pylzowi*, *P*. *vlangalii nanschanica*, *P*. *theobaldi theobaldi*, *P*. *theobaldi orientalis*, *P*. *erythrurus erythrurus* and *P*. *erythrurus prava*) could be found on the Qinghai-Tibetan Plateau (QTP) [30–33]. Recently, the toad-headed lizard genus *Phrynocephalus* has drawn the attention of physiological and genetic researchers for its adaptation of broad geographical areas (about 2200–5000 m above the sea level) [33]. Red tail toad-headed lizard (*P*. *erythrurus*) is considered to be the highest living reptiles in the world (mostly 4500–5000 m above sea level) [34]. Previous study showed that inhibited metabolic, lower anaerobic metabolism, elevated mitochondrial efficiency and a possible higher utilization of fat may effectively compensate for the negative influence of cold and low PO_2_ in *P*. *erythrurus* [35].

In this study, two closely related reptile species based on the biological evolution and phylogeny, *P*. *erythrurus* and *P*. *przewalskii* (mostly 1000–1500 m above sea level) were chosen to analyze the physiological and genetic characteristics of the highest living lizard in the following aspects: (1) evaluating oxygen transportation capacity through analyzing the degree of changes in hematological parameters; (2) preliminarily understanding the sequence divergence and expression of α- and β-like globin genes in these two species; (3) analyzing the structural stability of potentially T-state isoHbs by equilibrium MD simulations. This study may provide important information and new insights into the adaptive mechanism of high-altitude ectothermic vertebrates.

## Materials and Methods

### Animals and sampling

All experiments were carried out according to protocols approved by the Ethics Committee of Animal Experiments at Lanzhou University and in accordance with guidelines from the China Council on Animal Care. *Phrynocephalus erythrurus* with an average weight of 6.69 ± 0.13 g were captured by hand in the wild at Tuotuo River (34°13'N, 92°13'E, 4543 m above sea level), Qinghai province, China, and *P*. *przewalskii* (the low-altitude sample) with an average weight of 6.92 ± 0.14 g were collected from a semi-desert areas in Minqin (38°38'N, 103°05'E, 1482 m above sea level), Gansu province, China. The Hoh-xil National Nature Reserve and Minqin Desert Control Station are only used for scientific research and the two authorities permitted us to capture the animals used in this study. Our studies did not involve endangered or protected species. All surgery was performed under sodium pentobarbital anaesthesia. Every effort was made to minimize the numbers used and any suffering experienced by the animals in the experiments.

Total 45 adult male lizards of each species were used in this study. Blood samples were obtained from the aortic arch directly in freshly anaesthetized lizard using a heparinized glass capillary tube. After blood collection, both liver and skeletal muscle were harvested by surgery and blotted with filter paper to remove excess liquid. The amount of blood taken from each animal was typically around 150 μL. Blood samples for hematological parameters (n = 12, 50 μL), blood gas (n = 12, 120 μL) and organic phosphate (ATP, n = 12, 150 μL) were placed immediately on ice and were measured within 1–2 hour nearby the capture location. Blood samples for the reverse-phase high performance liquid chromatography (RP-HPLC) (n = 12, the remaining 100 μL of hematological measurements) and the liver and skeletal muscle for the rapid amplification of cDNA ends (RACE) PCR (n = 36, all blood collected lizards) were immediately frozen in liquid nitrogen, and then stored at -80°C prior to use.

Nine lizards of each species from the collecting zone were brought to the laboratory at Lanzhou University (36°05'N, 103°86'E) within 48 hours of capture. High- and low-altitude lizards were maintained in an air-conditioned room with two self-contained non-pressurized hypoxic chambers (100 cm length, 45 cm width and 45 cm height). In order to minimize the possible effect of changed environments, conditions of chambers were set up to equivalent altitude of 4550m and 1450m (temperature, 16±0.5°C, 35±0.5°C, respectively, using 60 W bulbs and an air-conditioning system; PO_2_, ~92 and ~137 mmHg, respectively, using mixed gas of nitrogen and atmosphere; light: dark, 12h: 12h, using fluorescent lamps; food and water ad libitum) [35]. Blood samples were obtained using above-mentioned method. The determination of blood-O_2_ affinity was finished within 4 days of the collection. After sampling, all lizards were sacrificed with an overdose of barbiturate.

### Hematological parameters

Hemoglobin concentration ([Hb]) was measured by mixing 10 μL of blood with 2.5 mL of Van Kampen-Zijlstra solution and a spectrophotometer (Unico UV-2000) at the wave length of 540 nm. Red blood cell (RBC) count was measured by mixing 10 μL of blood into 1.99 mL RBC diluents and the count of erythrocytes was made in hemocytometer under microscope. Hematocrits (HCT) were determined by a modified Guest-Siler (1934) technique [36]; and erythrocyte diameters were measured on dried smears with an ocular micrometer.

### Arterial blood gas analysis

Arterial blood gas and major inorganic ions were measured by a blood gas analyzer (OPTI CCA-TS Analysator, OPTI Medical System Inc., Roswell, GA) [37] with a ComfortSampler arterial blood gas collection kit and type E-Cl BP7559 cassettes. Total 120 μL blood sample was used for arterial blood gas analysis according to the manufacturer's instructions.

### Oxygen dissociation curve and the concentration of ATP in erythrocytes

Oxygen dissociation curves were determined using a Hemox-Analyser (TCS Scientific Corp., USA). Total 40 μL blood sample from each lizard was dissolved in 3.96 mL buffer solution which contained 200 μL NaCl (3 mol/L), 40 μL 10% bovine serum albumin (BSA), 400 μL HEPES (0.5 mol/L, pH = 7.3), 40 μL anti-foaming agent and 3.34 mL distilled water. RBCs remained intact throughout the measurement procedure. All samples were analyzed at the temperature of 30°C. The gas mixtures used were 2% CO_2_ in air to establish full oxygenation and pure nitrogen for deoxygenation. OECs were directly plotted by software provided with the Hemox-Analyser. P_50_ were also obtained from this software. The concentration of ATP in erythrocytes was measured using ATP kits (Nanjing Jiancheng Bioengineering Institute, Jiangsu, China).

### RP-HPLC analysis

Samples were prepared from hemolysate with an Hb concentration of 40 g/L and were diluted further with water (75 μL hemolysate plus 925 μL water). Total 20 μL of diluted samples were used for each assay. Bio-Bond C4 column (5 μm, 250 x 4.6 mm, DIKMA) was used for RP-HPLC analysis. We eluted globin chains with a two-solvent system [solvent A, 200 mL/L acetonitrile and 3 mL/L trifluoroacetic acid (TFA) in water; solvent B, 600 mL/L acetonitrile and 3 mL/L TFA in water] and a 3-step RPLC elution program consisting of a linear gradient of 60%–100% solvent B in 80 min, a linear gradient of 100%–60% solvent B in 10 min, and reequilibration with 60% solvent B for 10 min. The flow rate was 1 mL/min, eluate was detected at 220 nm [38] and abundance were quantified using Image J [39]. Molecular weight of globins were detected using a MaXis 4G ultra-high resolution time of flight mass spectrometer (Bruker-Daltonics).

### RNA isolation, cDNA synthesis and RACE amplification

Total RNA was extracted and purified from liver and muscle of both two species. RNA concentration and purity was assayed using the NanoDrop 2000 (Thermo Scientific, USA). The integrity of the RNA was confirmed using electrophoresis. Full length cDNA for α- and β-globin genes was performed using a SMART RACE cDNA Amplification Kit (Clontech Laboratories) and the residue of genomic DNA was executed using Recombinant DNase I according to the manufacturer’s instruction. The primers were designed based on the sequences of *Anolis carolinensis* obtained from GenBank as shown in Table 1. The PCR amplification was performed using Touchdown PCR and LA Taq polymerase. PCR products were cloned into pMD18-T Vector (Takara, Dalian, China) and sequenced (Sangon, Shanghai, China). The sequences were deposited into GenBank (Accession number: KP019961-KP019968).

**Table 1:** Primer sequence used for RACE amplification.

Primers name	Primer sequence(5’-3’)
Hbα-F	GCTGCGGGTGGACccngknaaytt
Hbα-R	TAACGGTAYTTGGMGGTCAGCACRG
Hbβ-F	ATGGTGCACTGGACCGCCGAAGA
Hbβ-R	TCAGTGGTACCGGCGGGACAGG

### Preparation of Hb models and simulation setup

Primary structures of the α- and β-globin polypeptides were deduced from translated DNA sequences and there are potentially three different isoforms in each species. We used MODELLER 9v12 [40] to construct Hb tetramer models in the two species using the *Homo sapiens* deoxyhemoglobin (T-state) 1BZZ as a structural template. Total six Hb models were constructed and the α- and β-globin subunits composition in the two species as show in Table 2. Missing hydrogen atoms were added by the psfgen plugin of VMD [41]. The starting models were immersed in equilibrated TIP3P water boxes. To reflect physiological salt concentrations, NaCl were added to all the six systems (0.17 mol/L NaCl in *P*. *przewalskii*, 0.16 mol/L NaCl in *P*. *erythrurus*) using the autoionize plugin of VMD. The total system sizes were almost the same as 5003 atoms with an initial simulation box of 87 × 77 × 79 Å^3^ (hwHb1 of *P*. *erythrurus*).

**Table 2:** α- and β-globin subunits composition and named of the six Hb models.

		group 1	group 2	group 3
*P*. *przewalskii*	Composition	(αβ1)2	(αβ2)2	(αβ3)2
	Named	hsHb1	hsHb2	hsHb3
*P*. *erythrurus*	Composition	(αβ1)2	(αβ2)2	(αβ3)2
	Named	hwHb1	hwHb2	hwHb3

All simulations were performed with NAMD2.9 [42] using CHARMM version c35b2 with the all-atom 27 protein force field. The intramolecular bonds involved hydrogen atoms were constrained using the SHAKE algorithm, allowing a 2 fs integration time step. The energy minimizations were performed before the equilibration runs. Waters were melted while others were fixed for 500 ps period, this was followed by 500 ps runs with protein. After they were released, the system was subjected to equilibration runs for 10.5 ns. Simulations were performed with controlling of the constant pressure temperature (P = 1 atm, T = 310 K), Periodic boundary conditions were applied, and the electrostatic interactions were calculated by the particle-mesh Ewald method. After simulations, all analysis was used VMD and corresponding Plugs within the final 4 ns.

### Statistical analyses

The data on hematology, blood gas, P_50_ and ATP concentration were test for normality and homogeneity of the variances before ANOVA. Then data were analyzed using one-way analysis of variance (ANOVA). Values presented as mean ± SEM, statistical significance was accepted at P < 0.05.

## Results

### Hematological parameters and blood gas analysis

The experimental measures of hematological parameters under habitat conditions of both species are presented in Table 3. High-altitude *P*. *erythrurus* exhibits elevated RBC (1.12±0.04 and 0.94±0.04 × 10^12^/L, respectively; F_1, 23_ = 28.36, p<0.05), [Hb] (107.92±4.32 and 92.48±2.88 g/L, respectively; F_1, 20_ = 19.86, p<0.01) and Hematocrit (HCT, 32.82±1.10 and 27.70±0.47%, respectively; F_1, 23_ = 25.25, p<0.001) compared with the average values of low-altitude *P*. *przewal*skii. There was no significant variation in the average values for mean corpuscular hemoglobin concentration (MCHC, 339.75±15.68 and 328.13±13.31 g/L, respectively; F_1, 14_ = 0.319, p>0.05), mean corpuscular volume (MCV, 0.27±0.01 and 0.31±0.02 pL, respectively; F_1, 14_ = 2.582, p>0.05) and mean cell hemoglobin (MCH, 91.41±5.46 and 100.20±5.92 pg, respectively; F_1, 14_ = 1.192, p>0.05) between these two species. Blood gas analyzer was applied to the small reptile for the first time, and the results are presented in Table 4. Oxygen partial pressure (PaO_2_, 56.38±1.53 and 77.28±2.72 mmHg, respectively; F_1, 22_ = 44.90, p<0.001), carbon dioxide partial pressure (PaCO_2_, 27.83±2.20 and 38.13±2.83 mmHg, respectively; F_1, 22_ = 8.23, p<0.01), arterial oxygen saturation (SaO_2_, 77.47±1.11 and 83.96±1.40%, respectively; F_1, 22_ = 13.16, p<0.01), [HCO_3_^-^] (14.30±1.07 and 18.86±1.16 mmol/L, respectively; F_1, 20_ = 5.50, p<0.05), [Na^+^] (159.09±1.14 and 169.38±1.57 mmol/L, respectively; F_1, 20_ = 28.03, p < 0.001) and [Cl^-^] (117.57±1.11 and 123.44±1.02 mmol/L, respectively; F_1, 20_ = 15.17, p<0.01) in arterial blood of *P*. *erythrurus* were significantly lower than that in *P*. *przewalskii*, while pH (7.33±0.03 and 7.31±0.03, respectively; F_1, 22_ = 0.17, p>0.05) and [K^+^] (4.00±0.15 and 4.39±0.29 mmol/L, respectively; F_1, 22_ = 1.33, p>0.05) have no significant variation between the species.

**Table 3:** Hematological parameters of *P*. *erythrurus* and *P*. *przewalskii*.

Determination	*P*. *przewalskii*	*P*. *erythrurus*	p-Value
RBC count × 10^12^/L	0.94±0.04	1.12±0.04	0.029
Hematocrit (%)	27.70±0.47	32.82±1.10	0.010
Hemoglobin (g/L)	92.48±2.88	107.92±4.32	0.002
MCV (pL)	0.31±0.02	0.27±0.01	0.134
MCH (pg)	100.20±5.92	91.41±5.46	0.293
MCHC (g/L)	328.13±13.31	339.75±15.68	0.581

Data presented as mean ± SEM; RBC, red blood cell; MCV, mean corpuscular volume; MCH, mean cell hemoglobin; MCHC, mean corpuscular hemoglobin concentration.

**Table 4:** Arterial blood gas measurements of *P*. *erythrurus* and *P*. *przewalskii*.

Determination	*P*. *przewalskii*	*P*. *erythrurus*	p-Value
pH	7.31±0.03	7.33±0.03	0.670
PaCO_2_ (mmHg)	38.13±2.83	27.83±2.20	0.009
PaO_2_ (mmHg)	77.28±2.72	56.38±1.53	0.000
HCO_3_^-^(mmHg)	18.86±1.16	14.30±1.07	0.028
Na^+^ (mmol/L)	169.38±1.57	159.09±1.14	0.000
K^+^ (mmol/L)	4.39±0.29	4.00±0.15	0.262
Cl^-^ (mmol/L)	123.44±1.02	117.57±1.11	0.001
SaO_2_ (%)	83.96±1.40	77.47±1.11	0.001

Data presented as mean ± SEM; PaO_2_, arterial blood oxygen partial pressure; PaCO_2_, arterial blood carbon dioxide partial pressure; SaO_2_, arterial blood oxygen saturation.

### Whole blood Oxygen affinity and the concentration of ATP in erythrocytes

**Fig 1:**
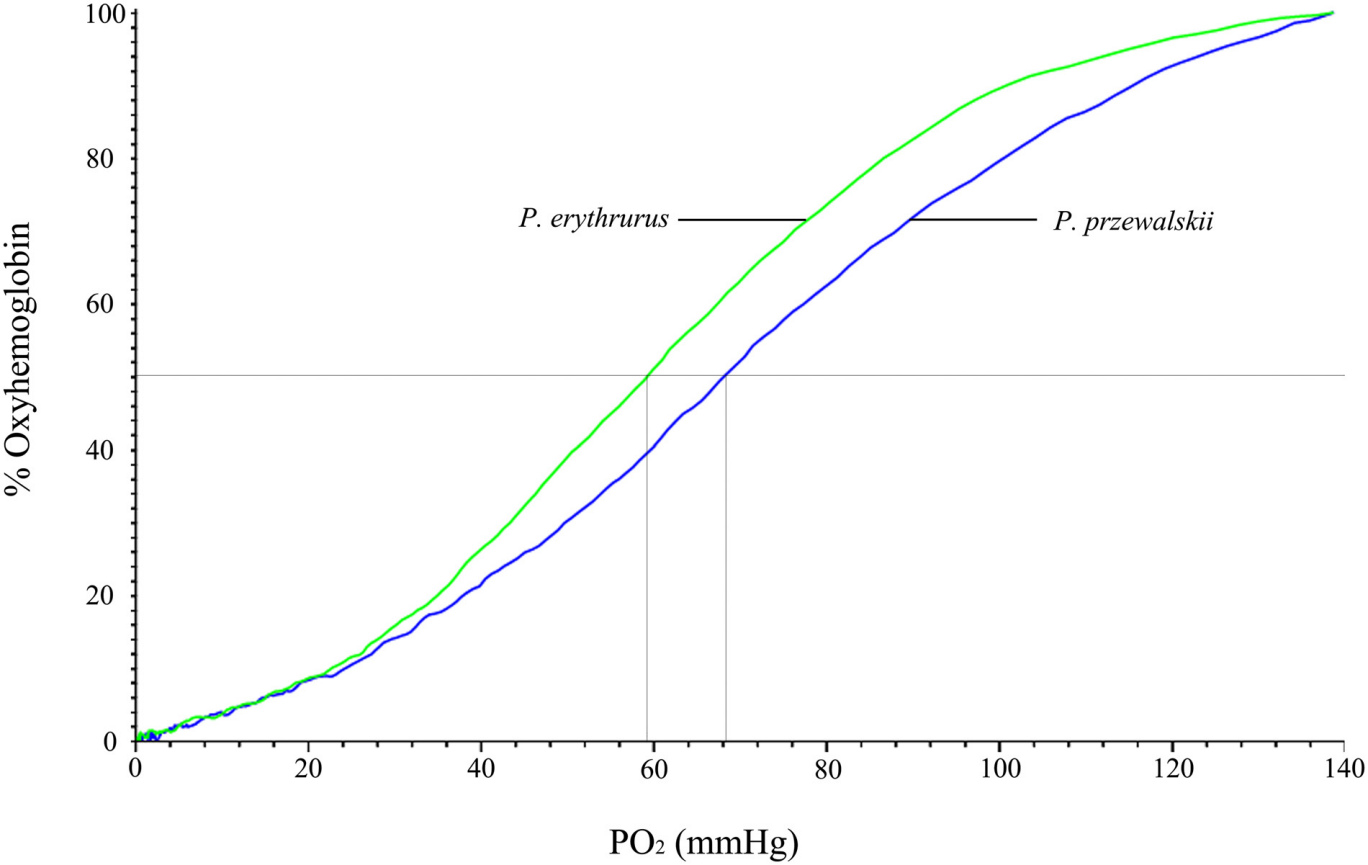
Whole blood oxygen dissociation curves of *P*. *erythrurus* (green line) and *P*. *przewalskii* (blue line) at 30°C and pH 7.3.

Oxygen equilibrium measurements of the whole blood showed that *P*. *erythrurus* (green line) exhibits a higher O_2_ affinity compared with *P*. *przewalskii* (blue line) under 30°C and pH 7.3 (Fig 1). There were significant differences in P_50_ (51.97±2.64 and 71.27±1.49 mmHg, respectively; F_1, 20_ = 49.66, p<0.001) and ATP concentration (200.52 and 91.33 μmol/gHb, respectively; F_1, 20_ = 50.68, p<0.05) between these two species, and ATP concentration exhibits two-fold correlation between them (Table 5). However, we did not detect any difference in oxygen affinity between the sexes in both species.

**Table 5:** ATP concentration and P_50_ of *P*. *erythrurus* and *P*. *przewalskii*.

Determination	*P*. *przewalskii*	*P*. *erythrurus*	p-Value
RBC [ATP] (μmol/gHb)	91.33±9.11	200.52±28.97	0.040
P_50_ (mmHg)	71.27±1.49	51.97±2.64	0.000

Data presented as mean ± SEM.

### RP-HPLC and amino acid sequence analysis

**Fig 2:**
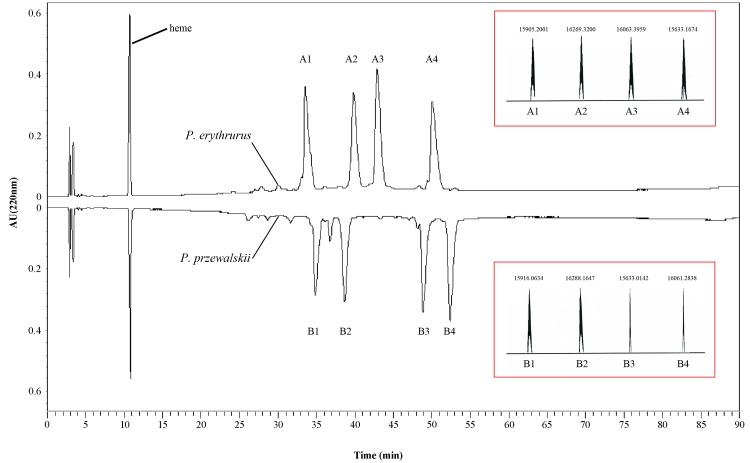
RP-HPLC chromatograms for erythrocyte hemolysates of *P*. *erythrurus* (upward profile) and *P*. *przewalskii* (downward profile). Four globin chain peaks of *P*. *erythrurus* (A1–A4) and *P*. *przewalskii* (B1–B4) were eluted from the C4 RP-HPLC column, and corresponding molecular weight was shown in a red box.

RP-HPLC analysis of hemolysate showed approximately equal amounts of globin peaks in these two species (Fig 2). There are four major peaks A1, A2, A3 and A4 (molecular weight, 15905.2001, 16269.3200, 16063.3959 and 15633.1674 Da, respectively) with a roughly abundance ratio 1.05: 1.04: 1.29: 1.11 in *P*. *erythrurus*. The four major peaks were also detected including B1, B2, B3 and B4 (molecular weight, 15916.0634, 16288.1647, 15633.0142 and 16061.2838 Da, respectively) with a abundance ratio 0.74: 0.87: 0.99: 1.05 in *P*. *przewalskii*. Intriguingly, a low-abundance peak (molecular weight, 15909.0636; relative abundance, 0.17) between B1 and B2 was detected in all analysis of *P*. *przewalskii*.

**Fig 3:**
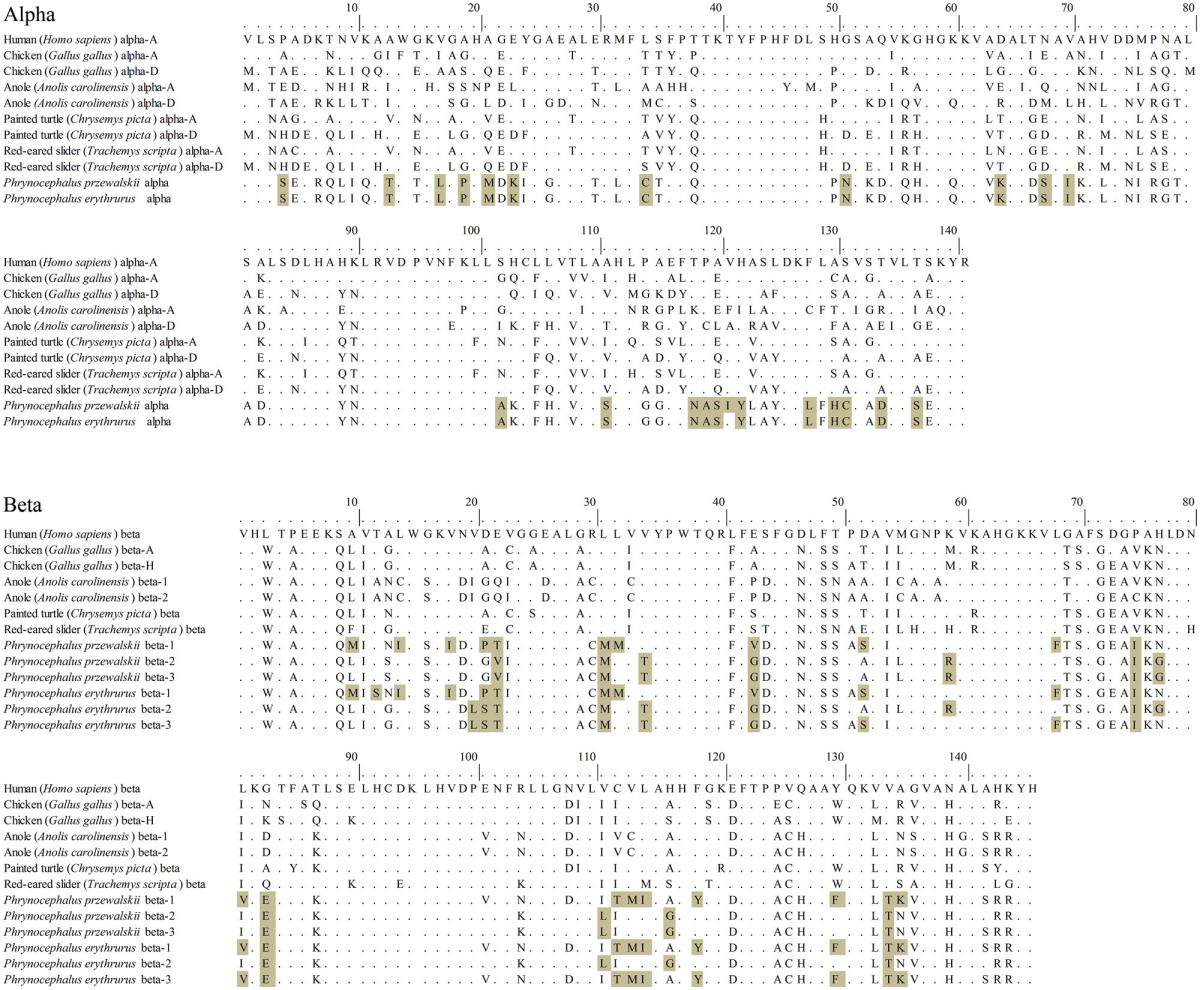
Alignment amino acid sequence of α- and β-like globin chains from the two lizard species and five outgroup taxa: human (*Homo sapiens*), chicken (*Gallus gallus*), anole lizard (*Anolis carolinensis*), red-eared slider (*Trachemys scripta*) and painted turtle (*Chrysemys picta*). The α- and β-globin polypeptides of both species were deduced from translated DNA sequences, peculiar amino acid of the two species were marked with light gray boxes.

One α-globin gene and three β-globin genes were cloned and sequenced in both species. The α- and β-globin polypeptides were deduced from translated DNA sequences. Alignment amino acid sequences of α- and β-like globin chains from these two lizard species and five outgroup taxa: human (*Homo sapiens*), chicken (*Gallus gallus*), anole lizard (*Anolis carolinensis*), red-eared slider (*Trachemys scripta*) and painted turtle (*Chrysemys picta*) were shown in Fig 3. Compared with these five taxa, total of 23 and 27 peculiar sites (Alpha and Beta, respectively) were found in these two lizard species. Furthermore, the α-globin chains of these two species are distinguished only by one amino acid in position of 121 (Ile-Val). For the multiple alignment, a total of 33 site differences were discovered. Then we detected varying degrees of amino acid sequence difference in β1-, β2- and β3-globin chains between these two species (the number of amino acid substitutions are 1, 5 and 24 respectively). The results showed that the β1-globin chain has the highest sequence identity with only one substitution (β12Thr-Ser). Identical substitutions of β-globin chains in these two species occur in position β10, β14, β18, β29, β32, β34, β43 and β142. There was one particular substitution at site β22 (Thr-Val) in *P*. *przewalskii*, meanwhile five particular substitutions at sites β12 (Thr-Ser), β13 (Asn-Gly VS Asn-Ser in *P*. *przewalskii*), β20 (Val-Leu), β21 (Pro-Ser VS Pro-Gly in *P*. *przewalskii*), β23 (Ile-Val) were found in *P*. *erythrurus*.

### Structural and dynamical analysis of the six Hb models

**Fig 4:**
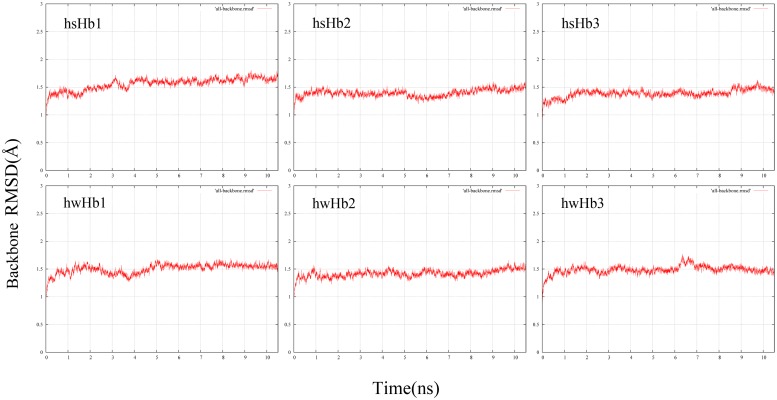
Evolution of root mean square deviation (RMSD) of the atoms in the backbone of the proteins over time from the initial structure for: *P*. *przewalskii* (hsHb1, hsHb2, hsHb3) and *P*. *erythrurus* (hwHb1, hwHb2, hwHb3).

**Fig 5:**
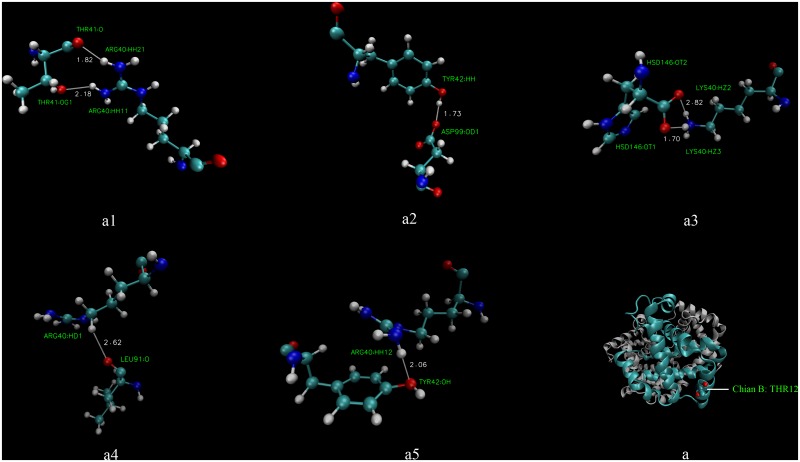
The loss of 7 hydrogen bonds at α2β1 interface in hwHb1. (a1–a5) Hydrogen bonds at α2β1 interface present in hsHb1 and lost in hwHb1, (a) Three-dimensional structure of hsHb1 with β12Met.

**Fig 6:**
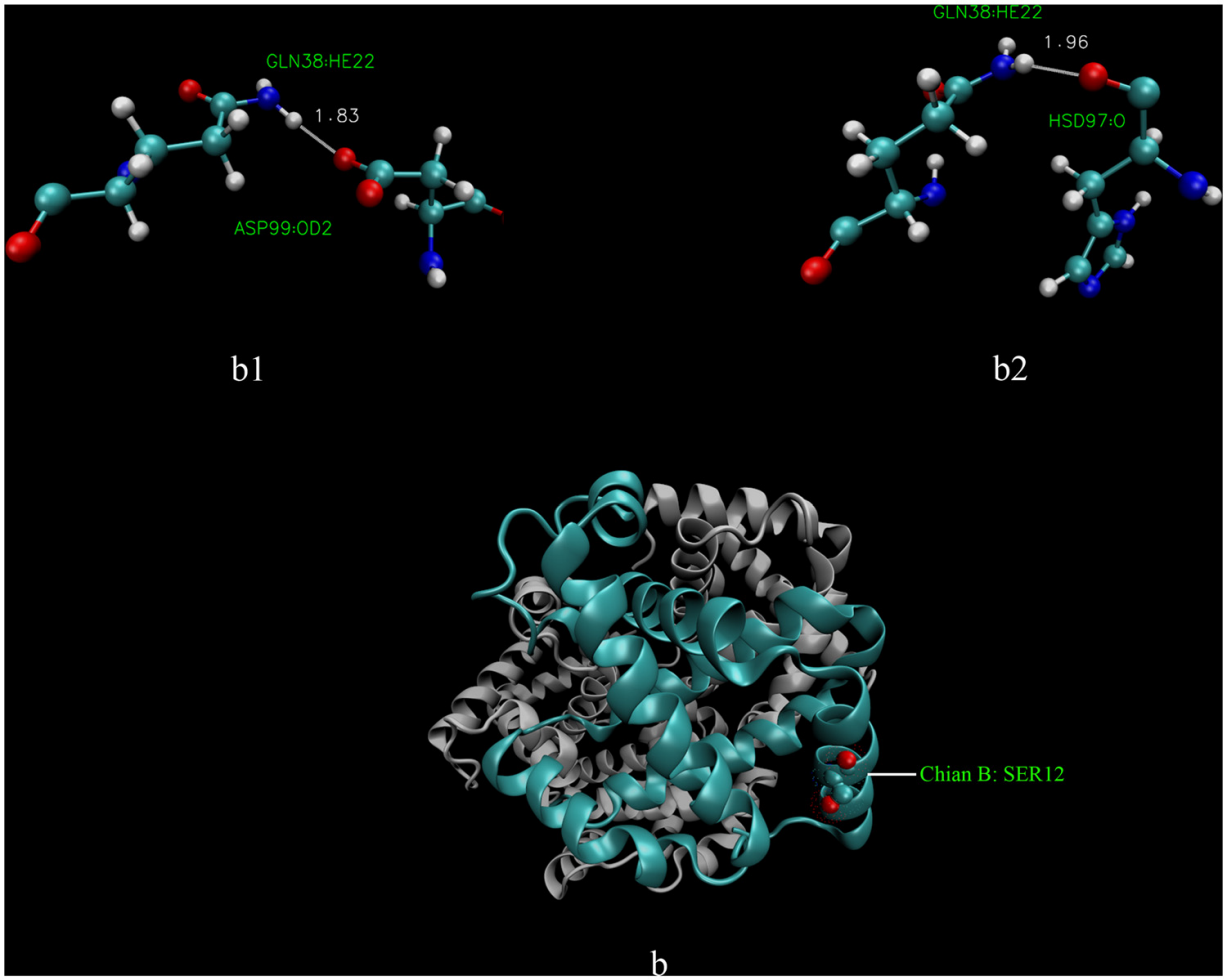
The reformed of 2 hydrogen bonds at α2β1 interface in hwHb1. (b1–b2) Hydrogen bonds at α2β1 interface present in hwHb1 and lost in hsHb1, (b) Three-dimensional structure of hwHb1 with β12Ser.

We employed equilibrium MD simulations on the six possible α_2_β_2_ tetrameric (hsHb1, hsHb2, hsHb3, hwHb1, hwHb2 and hwHb3) to observe the stability of T-state isoHbs. It was obvious that there are no large structural fluctuations during all the six simulations, as evidence in the time evolution of the backbone root mean square deviation (RMSD) shown in Fig 4. All the six models reach equilibrium within the first 5 ns of the simulations, and average backbone RMSD values were approximately 1.5 Å, so we analyzed the intersubunit contacts in the final 4 ns of the trajectories. Hydrogen bonds and salt bridges at α1β2 and α2β1 interfaces of the six Hb models are shown in Table 6. Total of 24, 28 and 32 hydrogen bonds were found in hsHb1, hsHb2 and hsHb3 of *P*. *przewalskii* respectively, and hydrogen bonds in *P*. *erythrurus* (19, 23, 27 in hwHb1, hwHb2, hwHb3, respectively) were significantly less than that in *P*. *przewalskii*. We found one salt bridge between α94Asp and β40Arg in all the six Hb models except in hsHb3 which formed an additional salt bridge between α40Lys and β94Asp at α2β1 interfaces. In group 1, there are 11 hydrogen bonds at α1β2 interface in both models, simultaneously, the lost 7 hydrogen bonds (Fig 5) and reformed 2 hydrogen bonds at α2β1 interface were found in hwHb1 of *P*. *erythrurus* compared with hsHb1 of *P*. *przewalskii* (Fig 6). Similar results were also found in group 2 and group 3 with the lost 5 hydrogen bonds in isoHb models of *P*. *erythrurus* compared with *P*. *przewalskii* (data not shown).

**Table 6:** Hydrogen bonds and salt bridges at α1β2 and α2β1 interfaces of the six Hb models.

		hsHb1	hsHb2	hsHb3	hwHb1	hwHb2	hwHb3
Hydrogen bonds	α1β2	11	18	19	11	14	15
	α2β1	13	10	13	8	9	11
	total	24	28	32	19	23	27
Salt bridges	α1β2	1	1	1	0	0	0
	α2β1	0	0	1	1	1	1
	total	1	1	2	1	1	1

## Discussion

Matching O_2_ supply with O_2_ demand has always been a hot topic in the studies of high altitude adaptation. The highest living lizard *P*. *erythrurus* has to manage an unremitting hypobaric hypoxia (atmospheric pressure, 587.79 hPa; PO_2_, ~92 mmHg) and cold temperatures while *P*. *przewalskii* lives in a relatively mild environment (atmospheric pressure, 863.73 hPa; PO_2_, ~136 mmHg) [35]. The present study offers a snap-shot of hematological characteristics in these two lizard species dwelling at different altitudes for the first time. Our results indicated that *P*. *erythrurus* has an efficient oxygen transport system by regulating several steps in the O_2_ cascade.

When lowland natives ascend to high altitude, many of them can compensate for a reduced O_2_ supply by increasing their Hct, [Hb] and RBC. However, an excessive increased Hct will increase blood viscosity and add more budgets for heart, pulmonary and blood circulation system [15,43]. In humans, the available evidence indicates that the optimal Hb concentration at high altitude should be maintained at the typical sea level value and the hypoxia-induced polycythemia is a maladaptive plasticity [1,44]. A moderate increased Hct and [Hb] can be propitious to increase blood O_2_-carrying capacity and to improve tissue oxygenation which must be closer to the optimal values. Comparing our data to available hematologic values in mammals and birds, we found that [Hb] and Hct of both lizards were slightly lower than values reported previously. The optimal Hct for O_2_ transport was 40% in dogs [45,46]. [Hb] and hematocrit in imprisoned bar-headed goose (Anser indicus) were 17.1±1.24 g/dL and 43.3±3.9%, respectively [47]. In addition, hematological observations have been reported in several reptiles from sea level to 3350 m including RBC (range from 0.955 to 1.37 × 10^12^/L), Hct (25 to 39%) and [Hb] (67 to 114 g/L) [48–51]. The values of both species in this study intervene between the minimum and maximum values of reported reptiles. Meanwhile, Hct of *P*. *erythrurus* is very close to the calculated optimal values for balancing O_2_ carrying capacity and blood viscosity from the lizard *Dipsosaurus dorsalis*, which break the bonds of convention in most small lizards (body mass <8 g, Hct < 30%) [52]. Unlike the hypoxia-induced maladaptive polycythemia, the elevation of RBC in *P*. *erythrurus* could promote oxygen carrying capacity without disadvantage of high viscosity. In addition, our previous study indicated a closely related species *P*. *vlangalii* can increase its oxygen carrying capacity in hypoxic acclimatization and adaptation [53]. When acclimatized to environmental hypoxia low-altitude *P*. *vlangalii* exhibited unchanged RBC and elevated Hct and [Hb], MCV and MCHC and similar result was obtained when comparing these parameters in *P*. *vlangalii* living at different altitudes. Our results showed a more propitious Hct in *P*. *erythrurus* compared to high-altitude *P*. *vlangalii* and a different strategy for *P*. *erythrurus* to increase oxygen transport efficiency by increasing RBC rather than increasing the volume of red blood cell.

The PaO_2_ largely mirror the effectiveness of ventilation and pulmonary diffusion with hypoxia [1,6]. The ambient oxygen partial pressure descend from 136 to 92 mmHg from 1500 m to 4500 m altitude while the PaO_2_ of *P*. *przewalskii* and *P*. *erythrurus* descend from about 77 to 56 mmHg. Furthermore, a lower PaO_2_ in *P*. *erythrurus* does not caused the secondary alkalosis by accelerated breathing. This result suggest that *P*. *erythrurus* may have been evolved an efficient pulmonary system for O_2_ loading during the prolonged hypoxia. The lower PaCO_2_ of *P*. *erythrurus* may be due to the suppressed aerobic metabolism [35]. The blunted hypoxic ventilatory response in *P*. *erythrurus* might help to reduce the oxygen cost of breathing and respiratory water loss [6].

Fine-tuned adjustments in blood-O_2_ affinity play an important role in matching O_2_ supply and O_2_ demand under high altitude hypoxia. Our result indicated that *P*. *erythrurus* has an elevated blood-O_2_ affinity compared with low-altitude *P*. *przewalskii*. This may be achieved by changes in intrinsic Hb-O_2_ affinity, the sensitivity of Hb to allosteric cofactors and the concentration of allosteric cofactors. Firstly, Our results demonstrated multiple substitutions of amino acid in Hb. Certain residues from human Hb have been demonstrated for proton binding (α1Val, α122His, β2His, β82Lys, β143His, and β146His), chloride ions binding (α1Val and α131Ser and one β1Val and β82Lys) and CO_2_ binding (N-terminal NH3^+^ residues) [54–56]. However, checking these three potential binding sites, we did not found any substitutions between two lizard species. Besides, ATP binding site has been described in Hbs of fish including β1Val, β2Glu, β82Lys and β143Arg [57]. We found that β-globin of both lizards contains His at β2, but this change may not alter the responsiveness to ATP based on the evidence reported in red-eared slider [27]. Consequently, the elevated blood-O_2_ affinity in *P*. *erythrurus* could not be caused by the change in sensitivity of Hb to allosteric cofactors. Secondly, the concentration of ATP in erythrocytes in *P*. *erythrurus* is over twice than that in *P*. *przewalskii*. Conversely, [Cl^-^], [HCO_3_^-^] and PaCO_2_ in blood of *P*. *erythrurus* were significantly lower. However, no significant variation of pH was found between these two species. These results suggest that the elevated blood-O_2_ affinity in *P*. *erythrurus* may be attributable to balancing the independent effects of these potential heterotropic ligands under the prevailing conditions. Finally, amino acid substitutions that located at α1β2 and α2β1 interfaces of the isoHbs may be critical for controlling Hb-O_2_ affinity by impact the transformation process from the T-state to the R-state during oxygenation of hemoglobin [18,58,59]. Our results suggest that isoHbs of *P*. *erythrurus* may have higher intrinsic Hb-O_2_ affinity compared with *P*. *przewalskii* which may due to the eliminated hydrogen bonds at α1β2 and α2β1 interfaces. Structural analysis shows that 2 of these 33 substitutions occurred at α1β2 or α2β1 interfaces including β34Val-Thr (nonpolar-polar) and β101Val-Glu (nonpolar-polar). These substitutions are conducive to form hydrogen bonds with α141Arg and α41Thr. The specific substitutions in position of β13(A9)Gly-Ser was also reported in Andean hummingbirds which increased O_2_-affinity in the presence of β83Gly and reduced O_2_-affinity in the presence of β83Ser (epistasis for Hb-O_2_ affinity) [20]. The position β83 of both *Phrynocephalus* lizards was occupied by Gln and the polarity of Gln is obviously closer to Ser. Therefore, this substitution may typically reduce O_2_-affinity of isoHb in *P*. *przewalskii*. In addition, the similar substitutions in position of β142 have been verified leading to increase in oxygen affinity [60]. All of these examples suggest a higher intrinsic O_2_ affinity of isoHbs in *P*. *erythrurus*. Hence, elevated blood-O_2_ affinity in *P*. *erythrurus* may mainly due to the higher intrinsic Hb-O_2_ affinity and concentration-dependent adjustment of allosteric cofactors.

As observed in many birds and nonavian reptiles, the phenomenon of co-express different isoHbs was also found in these two lizard species [20,27–29]. Although the function of each isoHb has not been confirmed, we can speculate that functionally distinct isoHbs exist in these two species. From number of hydrogen bonds in the six models, we can predict the oxygen affinity of isoHbs as follows: hwHb1 > hwHb2 > hsHb1 > hwHb3 > hsHb2 > hsHb3 (*P*. *erythrurus* > *P*. *przewalskii*). A potential mechanism for matching O_2_ supply with O_2_ demand in *P*. *erythrurus* could be provided by changes in intra-erythrocytic isoHbs stoichiometry [61–64]. All experiments of MD simulations in this study are based on identified one α-globin gene and three β-globin genes by RACE-PCR. There may be other homologous globin genes failed to be detected due to scarcity of available sequence in lizard species and increased sequence divergence in two distinct paralogs [25]. In sum, a variety of factors may lead to change of Hb-O_2_ affinity, future detailed studies on the relationship between structure and function of isoHbs in these two lizards may reveal novel molecular mechanisms of high altitude adaptation.

## Conclusion

As the highest living lizards in the world, *P*. *erythrurus* may have evolved an efficient oxygen transport system under an unremitting hypobaric hypoxia. It increases oxygen carrying capacity by increasing RBC and this could promote oxygen carrying capacity without disadvantage of high viscosity. The elevated blood-O_2_ affinity in *P*. *erythrurus* may be achieved by increasing in intrinsic O_2_ affinity of isoHbs and balancing the independent effects of potential heterotropic ligands.
